# 5,7,9,10-Tetra­hydro-5β,10β-methano-3aα,8aα-methyl­propenocyclo­octa­[1,2-*c*:5,6-*c*′]dipyrazole-3,8(2*H*,4*H*)-dione monohydrate

**DOI:** 10.1107/S1600536808013512

**Published:** 2008-05-10

**Authors:** Djamal Djaidi, Roger Bishop, Donald C. Craig, Marcia L. Scudder

**Affiliations:** a School of Chemistry, University of New South Wales, Sydney, Australia 2052

## Abstract

The racemic title compound, C_15_H_16_N_4_O_2_·H_2_O, crystallizes as a hydrogen-bonded layer structure incorporating the solvent water mol­ecules. Within the layers, there are three distinct hydrogen-bonding motifs which can be classified as *R*_2_^2^(8), *R*_4_^2^(8) and *R*_4_^4^(12).

## Related literature

For related literature, see: Chan *et al.* (2008 ▶); Yue *et al.* (1997 ▶, 2000 ▶, 2007 ▶). For hydrogen-bonding analysis, see: Etter (1990 ▶).
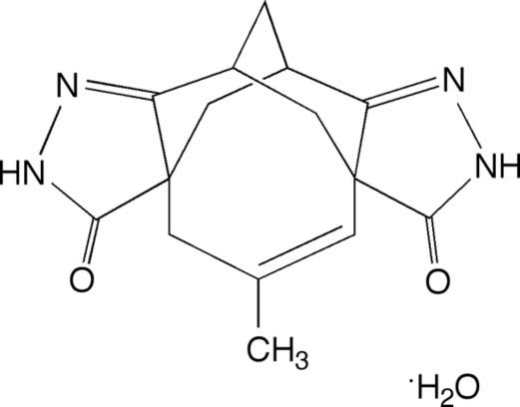


## Experimental

### 

#### Crystal data

C_15_H_16_N_4_O_2_·H_2_O
*M* *_r_* = 302.3
Triclinic, 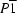

*a* = 6.478 (1) Å
*b* = 8.157 (1) Å
*c* = 14.812 (2) Å
α = 85.412 (9)°
β = 88.369 (8)°
γ = 67.089 (11)°
*V* = 718.6 (2) Å^3^
*Z* = 2
Cu *K*α radiation
μ = 0.82 mm^−1^
*T* = 294 K
0.30 × 0.25 × 0.22 mm

#### Data collection

Enraf–Nonius CAD-4 diffractometer
Absorption correction: none
2695 measured reflections
2695 independent reflections
2365 reflections with *I* > 2σ(*I*)
1 standard reflections frequency: 30 min intensity decay: none

#### Refinement

*R*[*F* ^2^ > 2σ(*F* ^2^)] = 0.045
*wR*(*F* ^2^) = 0.089
*S* = 1.64
2357 reflections
200 parameters
H-atom parameters not refined
Δρ_max_ = 0.34 e Å^−3^
Δρ_min_ = −0.22 e Å^−3^

### 

Data collection: *CAD-4* (Schagen *et al.*, 1989 ▶); cell refinement: *CAD-4*; data reduction: local program; program(s) used to solve structure: *SIR92* (Altomare *et al.*, 1994 ▶); program(s) used to refine structure: *RAELS* (Rae, 2000 ▶); molecular graphics: *ORTEPII* (Johnson, 1976 ▶) and *CrystalMaker* (CrystalMaker Software, 2005 ▶); software used to prepare material for publication: local programs.

## Supplementary Material

Crystal structure: contains datablocks I. DOI: 10.1107/S1600536808013512/tk2269sup1.cif

Structure factors: contains datablocks I. DOI: 10.1107/S1600536808013512/tk2269Isup2.hkl

Additional supplementary materials:  crystallographic information; 3D view; checkCIF report

## Figures and Tables

**Table 1:** Hydrogen-bond geometry (Å, °)

*D*—H⋯*A*	*D*—H	H⋯*A*	*D*⋯*A*	*D*—H⋯*A*
N2—H*N*2⋯O*W*^i^	1.00	1.83	2.763 (3)	154
N4—H*N*4⋯O2^ii^	1.00	2.00	2.858 (2)	143
O*W*—H1*OW*⋯O1	1.00	1.85	2.844 (2)	169
O*W*—H2*OW*⋯O1^iii^	1.00	1.81	2.796 (2)	169

Symmetry codes: (i) 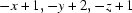; (ii) 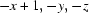; (iii) 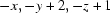.

**Fig. 1.**
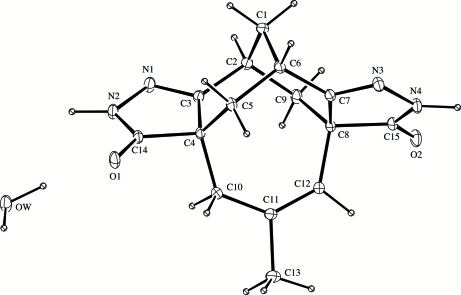
Molecular structure of (I) showing atom numbering scheme and dispacement ellipsoids drawn at the 30% probability level.

**Fig. 2.**
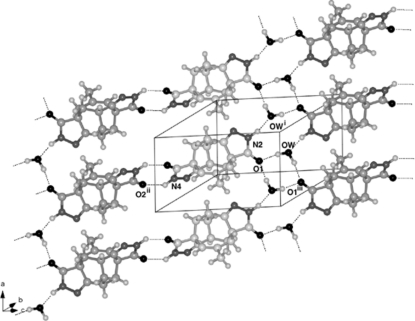
Part of one hydrogen bonded layer in the crystal structure of (I) showing the three hydrogen bonded packing motifs. Enantiomers are distinguished by C shading and hydrogen bonds are shown as dashed bonds.

## supplementary crystallographic information

### Comment

The structural core of the title compound (I) is the rare tricyclo[5.3.1.1^3,9^]dodecane ring system, the chemistry of which has been described by us earlier (Yue *et al.* 1997, 2000, 2007; Chan *et al.* 2008). Compound (I), Fig. 1, forms hydrogen bonded layers that lie in the (1 - 2 1) plane, Fig. 2 & Table 1. There are three motifs, all of which are centrosymmetric, which repeat within the layer. The first of these incorporates pairs of N—H···O=C hydrogen bonds. The second and third alternate along *a*, one comprising cycles of O—H···O=C hydrogen bonds and involving the lattice water molecules, and the other including N—H···O (water) interactions as well. In Etter's notation, the three cycles can be described as *R*_2_^2^(8), *R*_4_^2^(8) and *R*_4_^4^(12), respectively (Etter, 1990).

### Experimental

Racemic 3,7-bis(methoxycarbonyl)-5-methylidenetricyclo[5.3.1.1^3,9^]dodecane-2,8-dione (Yue *et al.*, 1997) (1.00 g, 3.24 mmol) was ground into a fine powder and then a small volume of hydrazine hydrate added. After stirring the mixture for 30 min, the resulting solid was filtered, washed with a small amount of diethyl ether and dried. The creamy material was recrystallized from methanol to give shiny crystals of the dipyrazole product (0.60 g, 68%), m.p. 335–343°C (decomp.). Found: C 61.90, H 6.24, N 20.97; C_15_H_16_N_4_O_2_.H_2_O requires C 61.75, H 5.93, N 20.58%. X-ray quality crystals were obtained from a methanol solution of (I).

### Refinement

Hydrogen atoms attached to C and N were included at calculated positions (C—H, N—H = 1.0 Å). The water hydrogen atoms were located on a difference map, and then positioned with O—H = 1.0 Å. All hydrogen atoms were refined with isotropic thermal parameters equivalent to those of the atom to which they were bonded. A small number of reflections were omitted from the refinement due to rounding differences between the data processing and refinement programs.

### Crystal data

**Table:**

C_15_H_16_N_4_O_2_·H_2_O	*Z* = 2
*M**_r_* = 302.3	*F*_000_ = 320.0
Triclinic, *P*1	*D*_x_ = 1.40 Mg m^−^^3^
Hall symbol: -P 1	Cu *K*α radiation λ = 1.54184 Å
*a* = 6.478 (1) Å	Cell parameters from 10 reflections
*b* = 8.157 (1) Å	θ = 20–25º
*c* = 14.812 (2) Å	µ = 0.82 mm^−^^1^
α = 85.412 (9)º	*T* = 294 K
β = 88.369 (8)º	Irregular, colourless
γ = 67.089 (11)º	0.30 × 0.25 × 0.22 mm
*V* = 718.6 (2) Å^3^	

### Data collection

**Table:**

Enraf–Nonius CAD-4 diffractometer	*h* = −7→7
ω–2θ scans	*k* = −9→9
Absorption correction: none	*l* = 0→18
2695 measured reflections	1 standard reflections
2695 independent reflections	every 30 min
2365 reflections with *I* > 2σ(*I*)	intensity decay: none
θ_max_ = 70º	

### Refinement

**Table:**

Refinement on *F*	H-atom parameters not refined
*R*[*F*^2^ > 2σ(*F*^2^)] = 0.045	*w* = 1/[σ^2^(*F*) + 0.0004*F*^2^]
*wR*(*F*^2^) = 0.089	(Δ/σ)_max_ = 0.003
*S* = 1.64	Δρ_max_ = 0.34 e Å^−^^3^
2357 reflections	Δρ_min_ = −0.22 e Å^−^^3^
200 parameters	Extinction correction: none

### Fractional atomic coordinates and isotropic or equivalent isotropic displacement parameters (Å^2^)

**Table:**

	*x*	*y*	*z*	*U*_iso_*/*U*_eq_	
O1	0.2515 (2)	0.7918 (2)	0.4385 (1)	0.0547 (4)	
O2	0.5206 (3)	0.2155 (2)	−0.00820 (9)	0.0554 (5)	
N1	0.7574 (3)	0.6073 (2)	0.3265 (1)	0.0467 (4)	
N2	0.6047 (3)	0.7257 (2)	0.3836 (1)	0.0444 (4)	
N3	0.3972 (3)	0.0850 (2)	0.2088 (1)	0.0454 (4)	
N4	0.4023 (3)	0.0817 (2)	0.1133 (1)	0.0450 (4)	
C1	0.7515 (3)	0.2116 (3)	0.3103 (1)	0.0473 (5)	
C2	0.7756 (3)	0.3578 (3)	0.2444 (1)	0.0423 (5)	
C3	0.6519 (3)	0.5264 (2)	0.2887 (1)	0.0357 (4)	
C4	0.4109 (3)	0.5822 (2)	0.3178 (1)	0.0327 (4)	
C5	0.3650 (3)	0.4204 (2)	0.3612 (1)	0.0389 (4)	
C6	0.5016 (3)	0.2421 (2)	0.3198 (1)	0.0398 (4)	
C7	0.4370 (3)	0.2210 (2)	0.2270 (1)	0.0355 (4)	
C8	0.4732 (3)	0.3268 (2)	0.1447 (1)	0.0344 (4)	
C9	0.6971 (3)	0.3513 (3)	0.1481 (1)	0.0420 (5)	
C10	0.2364 (3)	0.7009 (2)	0.2481 (1)	0.0407 (5)	
C11	0.1748 (3)	0.6505 (2)	0.1653 (1)	0.0399 (4)	
C12	0.2665 (3)	0.4969 (2)	0.1200 (1)	0.0414 (5)	
C13	−0.0245 (4)	0.7972 (3)	0.1192 (2)	0.0680 (7)	
C14	0.4063 (3)	0.7116 (2)	0.3883 (1)	0.0379 (4)	
C15	0.4741 (3)	0.2025 (2)	0.0724 (1)	0.0386 (4)	
OW	0.1914 (3)	1.1247 (2)	0.5054 (1)	0.0586 (5)	
HN2	0.6410	0.8123	0.4175	0.044	
HN4	0.3574	−0.0013	0.0801	0.045	
H1C1	0.8130	0.2158	0.3709	0.047	
H2C1	0.8364	0.0923	0.2867	0.047	
HC2	0.9377	0.3389	0.2415	0.042	
H1C5	0.2021	0.4462	0.3535	0.039	
H2C5	0.4019	0.4063	0.4272	0.039	
HC6	0.4903	0.1431	0.3614	0.040	
H1C9	0.6792	0.4657	0.1126	0.042	
H2C9	0.8150	0.2493	0.1194	0.042	
H1C10	0.2861	0.8001	0.2289	0.041	
H2C10	0.0935	0.7491	0.2830	0.041	
HC12	0.1872	0.4965	0.0631	0.041	
H1C13	−0.0602	0.7554	0.0622	0.068	
H2C13	−0.1567	0.8285	0.1606	0.068	
H3C13	0.0115	0.9048	0.1042	0.068	
H1OW	0.2311	1.0064	0.4799	0.059	
H2OW	0.0335	1.1680	0.5279	0.059	

### Atomic displacement parameters (Å^2^)

**Table:**

	*U*^11^	*U*^22^	*U*^33^	*U*^12^	*U*^13^	*U*^23^
O1	0.0468 (8)	0.0597 (9)	0.0644 (9)	−0.0226 (7)	0.0129 (7)	−0.0380 (7)
O2	0.086 (1)	0.0521 (8)	0.0354 (7)	−0.0325 (8)	0.0048 (7)	−0.0159 (6)
N1	0.0379 (8)	0.057 (1)	0.052 (1)	−0.0223 (7)	0.0050 (7)	−0.0231 (8)
N2	0.0428 (9)	0.0485 (9)	0.0496 (9)	−0.0229 (7)	0.0022 (7)	−0.0214 (7)
N3	0.058 (1)	0.0384 (8)	0.0434 (9)	−0.0206 (7)	0.0035 (7)	−0.0110 (6)
N4	0.059 (1)	0.0396 (8)	0.0417 (9)	−0.0229 (7)	0.0003 (7)	−0.0146 (6)
C1	0.043 (1)	0.038 (1)	0.047 (1)	0.0006 (8)	−0.0128 (8)	−0.0096 (8)
C2	0.0272 (8)	0.049 (1)	0.048 (1)	−0.0082 (7)	0.0010 (7)	−0.0206 (8)
C3	0.0312 (8)	0.0399 (9)	0.0380 (9)	−0.0143 (7)	0.0002 (7)	−0.0114 (7)
C4	0.0288 (8)	0.0329 (8)	0.0362 (9)	−0.0098 (6)	0.0000 (6)	−0.0135 (7)
C5	0.046 (1)	0.0374 (9)	0.0350 (9)	−0.0174 (8)	0.0046 (7)	−0.0101 (7)
C6	0.052 (1)	0.0319 (9)	0.0327 (9)	−0.0129 (8)	−0.0029 (7)	−0.0040 (6)
C7	0.0396 (9)	0.0294 (8)	0.0360 (9)	−0.0108 (7)	0.0010 (7)	−0.0073 (6)
C8	0.0405 (9)	0.0307 (8)	0.0312 (8)	−0.0116 (7)	−0.0004 (6)	−0.0090 (6)
C9	0.041 (1)	0.046 (1)	0.041 (1)	−0.0179 (8)	0.0087 (7)	−0.0176 (7)
C10	0.0388 (9)	0.0309 (9)	0.049 (1)	−0.0086 (7)	−0.0059 (8)	−0.0092 (7)
C11	0.0380 (9)	0.0348 (9)	0.0404 (9)	−0.0071 (7)	−0.0043 (7)	−0.0018 (7)
C12	0.047 (1)	0.0337 (9)	0.0417 (9)	−0.0115 (8)	−0.0104 (8)	−0.0063 (7)
C13	0.063 (1)	0.051 (1)	0.065 (2)	0.007 (1)	−0.023 (1)	−0.012 (1)
C14	0.0400 (9)	0.0360 (9)	0.0396 (9)	−0.0146 (7)	−0.0001 (7)	−0.0148 (7)
C15	0.046 (1)	0.0344 (9)	0.0350 (9)	−0.0133 (7)	−0.0017 (7)	−0.0107 (7)
OW	0.0515 (8)	0.062 (1)	0.074 (1)	−0.0306 (7)	0.0046 (7)	−0.0305 (8)

### Geometric parameters (Å, °)

**Table:**

O1—C14	1.229 (2)	C5—H1C5	1.000
O2—C15	1.229 (2)	C5—H2C5	1.000
N1—N2	1.402 (2)	C6—C7	1.491 (2)
N1—C3	1.284 (2)	C6—HC6	1.000
N2—C14	1.333 (2)	C7—C8	1.503 (2)
N2—HN2	1.000	C8—C9	1.542 (2)
N3—N4	1.415 (2)	C8—C12	1.534 (2)
N3—C7	1.283 (2)	C8—C15	1.531 (2)
N4—C15	1.342 (3)	C9—H1C9	1.000
N4—HN4	1.000	C9—H2C9	1.000
C1—C2	1.533 (3)	C10—C11	1.438 (3)
C1—C6	1.543 (3)	C10—H1C10	1.000
C1—H1C1	1.000	C10—H2C10	1.000
C1—H2C1	1.000	C11—C12	1.380 (3)
C2—C3	1.490 (2)	C11—C13	1.510 (3)
C2—C9	1.540 (3)	C12—HC12	1.000
C2—HC2	1.000	C13—H1C13	1.000
C3—C4	1.507 (2)	C13—H2C13	1.000
C4—C5	1.550 (2)	C13—H3C13	1.000
C4—C10	1.528 (2)	OW—H1OW	1.000
C4—C14	1.535 (2)	OW—H2OW	1.000
C5—C6	1.543 (2)		
			
N2—N1—C3	107.0 (1)	N3—C7—C6	121.8 (2)
N1—N2—C14	113.5 (1)	N3—C7—C8	113.9 (2)
N1—N2—HN2	123.2	C6—C7—C8	122.4 (2)
C14—N2—HN2	123.2	C7—C8—C9	112.6 (1)
N4—N3—C7	106.9 (2)	C7—C8—C12	112.7 (2)
N3—N4—C15	112.4 (1)	C7—C8—C15	99.0 (1)
N3—N4—HN4	123.8	C9—C8—C12	115.6 (2)
C15—N4—HN4	123.8	C9—C8—C15	112.0 (1)
C2—C1—C6	109.5 (1)	C12—C8—C15	103.3 (1)
C2—C1—H1C1	109.5	C2—C9—C8	114.2 (2)
C2—C1—H2C1	109.5	C2—C9—H1C9	108.3
C6—C1—H1C1	109.5	C2—C9—H2C9	108.3
C6—C1—H2C1	109.5	C8—C9—H1C9	108.3
H1C1—C1—H2C1	109.5	C8—C9—H2C9	108.3
C1—C2—C3	104.3 (2)	H1C9—C9—H2C9	109.5
C1—C2—C9	112.1 (2)	C4—C10—C11	127.5 (2)
C1—C2—HC2	108.2	C4—C10—H1C10	104.8
C3—C2—C9	115.7 (2)	C4—C10—H2C10	104.8
C3—C2—HC2	108.2	C11—C10—H1C10	104.8
C9—C2—HC2	108.2	C11—C10—H2C10	104.8
N1—C3—C2	120.9 (2)	H1C10—C10—H2C10	109.5
N1—C3—C4	113.8 (2)	C10—C11—C12	132.2 (2)
C2—C3—C4	122.7 (1)	C10—C11—C13	112.7 (2)
C3—C4—C5	111.0 (1)	C12—C11—C13	115.1 (2)
C3—C4—C10	115.8 (2)	C8—C12—C11	129.0 (2)
C3—C4—C14	98.9 (1)	C8—C12—HC12	115.5
C5—C4—C10	114.7 (1)	C11—C12—HC12	115.5
C5—C4—C14	111.9 (1)	C11—C13—H1C13	109.5
C10—C4—C14	103.2 (1)	C11—C13—H2C13	109.5
C4—C5—C6	114.4 (1)	C11—C13—H3C13	109.5
C4—C5—H1C5	108.2	H1C13—C13—H2C13	109.5
C4—C5—H2C5	108.2	H1C13—C13—H3C13	109.5
C6—C5—H1C5	108.2	H2C13—C13—H3C13	109.5
C6—C5—H2C5	108.2	O1—C14—N2	125.5 (2)
H1C5—C5—H2C5	109.5	O1—C14—C4	128.1 (2)
C1—C6—C5	111.6 (2)	N2—C14—C4	106.4 (1)
C1—C6—C7	103.6 (2)	O2—C15—N4	126.6 (2)
C1—C6—HC6	108.1	O2—C15—C8	127.0 (2)
C5—C6—C7	116.9 (1)	N4—C15—C8	106.3 (2)
C5—C6—HC6	108.1	H1OW—OW—H2OW	109.5
C7—C6—HC6	108.1		
			
C3—N1—N2—C14	5.0 (2)	C5—C4—C10—H2C10	62.5
C3—N1—N2—HN2	−175.0	C14—C4—C10—C11	178.2 (2)
N2—N1—C3—C2	−162.8 (2)	C14—C4—C10—H1C10	55.8
N2—N1—C3—C4	−0.5 (2)	C14—C4—C10—H2C10	−59.5
N1—N2—C14—O1	174.6 (2)	C3—C4—C14—O1	−175.7 (2)
N1—N2—C14—C4	−7.0 (2)	C3—C4—C14—N2	5.9 (2)
HN2—N2—C14—O1	−5.4	C5—C4—C14—O1	−58.8 (3)
HN2—N2—C14—C4	173.0	C5—C4—C14—N2	122.8 (2)
C7—N3—N4—C15	8.1 (2)	C10—C4—C14—O1	65.0 (2)
C7—N3—N4—HN4	−171.9	C10—C4—C14—N2	−113.4 (2)
N4—N3—C7—C6	−164.3 (2)	C4—C5—C6—C1	47.2 (2)
N4—N3—C7—C8	0.2 (2)	C4—C5—C6—C7	−71.8 (2)
N3—N4—C15—O2	170.8 (2)	C4—C5—C6—HC6	166.0
N3—N4—C15—C8	−12.5 (2)	H1C5—C5—C6—C1	167.9
HN4—N4—C15—O2	−9.2	H1C5—C5—C6—C7	49.0
HN4—N4—C15—C8	167.5	H1C5—C5—C6—HC6	−73.3
C6—C1—C2—C3	62.4 (2)	H2C5—C5—C6—C1	−73.5
C6—C1—C2—C9	−63.5 (2)	H2C5—C5—C6—C7	167.5
C6—C1—C2—HC2	177.4	H2C5—C5—C6—HC6	45.3
H1C1—C1—C2—C3	−57.6	C1—C6—C7—N3	108.8 (2)
H1C1—C1—C2—C9	176.5	C1—C6—C7—C8	−54.4 (2)
H1C1—C1—C2—HC2	57.4	C5—C6—C7—N3	−128.1 (2)
H2C1—C1—C2—C3	−177.6	C5—C6—C7—C8	68.8 (2)
H2C1—C1—C2—C9	56.5	HC6—C6—C7—N3	−5.9
H2C1—C1—C2—HC2	−62.6	HC6—C6—C7—C8	−169.0
C2—C1—C6—C5	−63.1 (2)	N3—C7—C8—C9	−125.5 (2)
C2—C1—C6—C7	63.5 (2)	N3—C7—C8—C12	101.6 (2)
C2—C1—C6—HC6	178.1	N3—C7—C8—C15	−7.0 (2)
H1C1—C1—C6—C5	56.9	C6—C7—C8—C9	38.9 (2)
H1C1—C1—C6—C7	−176.5	C6—C7—C8—C12	−94.0 (2)
H1C1—C1—C6—HC6	−61.9	C6—C7—C8—C15	157.4 (2)
H2C1—C1—C6—C5	176.9	C7—C8—C9—C2	−30.6 (2)
H2C1—C1—C6—C7	−56.5	C7—C8—C9—H1C9	−151.3
H2C1—C1—C6—HC6	58.1	C7—C8—C9—H2C9	90.1
C1—C2—C3—N1	105.6 (2)	C12—C8—C9—C2	100.9 (2)
C1—C2—C3—C4	−55.1 (2)	C12—C8—C9—H1C9	−19.8
C9—C2—C3—N1	−130.8 (2)	C12—C8—C9—H2C9	−138.4
C9—C2—C3—C4	68.4 (2)	C15—C8—C9—C2	−141.2 (2)
HC2—C2—C3—N1	−9.4	C15—C8—C9—H1C9	98.2
HC2—C2—C3—C4	−170.1	C15—C8—C9—H2C9	−20.5
C1—C2—C9—C8	45.0 (2)	C7—C8—C12—C11	69.6 (3)
C1—C2—C9—H1C9	165.7	C7—C8—C12—HC12	−110.4
C1—C2—C9—H2C9	−75.7	C9—C8—C12—C11	−61.8 (3)
C3—C2—C9—C8	−74.4 (2)	C9—C8—C12—HC12	118.2
C3—C2—C9—H1C9	46.3	C15—C8—C12—C11	175.5 (2)
C3—C2—C9—H2C9	164.9	C15—C8—C12—HC12	−4.5
HC2—C2—C9—C8	164.1	C7—C8—C15—O2	−172.1 (2)
HC2—C2—C9—H1C9	−75.2	C7—C8—C15—N4	11.2 (2)
HC2—C2—C9—H2C9	43.4	C9—C8—C15—O2	−53.2 (3)
N1—C3—C4—C5	−120.9 (2)	C9—C8—C15—N4	130.1 (2)
N1—C3—C4—C10	106.1 (2)	C12—C8—C15—O2	71.9 (2)
N1—C3—C4—C14	−3.3 (2)	C12—C8—C15—N4	−104.9 (2)
C2—C3—C4—C5	41.1 (2)	C4—C10—C11—C12	−9.2 (3)
C2—C3—C4—C10	−91.9 (2)	C4—C10—C11—C13	172.8 (2)
C2—C3—C4—C14	158.7 (2)	H1C10—C10—C11—C12	113.2
C3—C4—C5—C6	−33.8 (2)	H1C10—C10—C11—C13	−64.9
C3—C4—C5—H1C5	−154.5	H2C10—C10—C11—C12	−131.6
C3—C4—C5—H2C5	87.0	H2C10—C10—C11—C13	50.4
C10—C4—C5—C6	99.7 (2)	C10—C11—C12—C8	−0.9 (4)
C10—C4—C5—H1C5	−21.0	C10—C11—C12—HC12	179.1
C10—C4—C5—H2C5	−139.5	C13—C11—C12—C8	177.1 (2)
C14—C4—C5—C6	−143.1 (1)	C13—C11—C12—HC12	−2.9
C14—C4—C5—H1C5	96.1	C10—C11—C13—H1C13	−180.0
C14—C4—C5—H2C5	−22.4	C10—C11—C13—H2C13	−60.0
C3—C4—C10—C11	71.3 (2)	C10—C11—C13—H3C13	60.0
C3—C4—C10—H1C10	−51.0	C12—C11—C13—H1C13	1.6
C3—C4—C10—H2C10	−166.3	C12—C11—C13—H2C13	121.6
C5—C4—C10—C11	−59.9 (2)	C12—C11—C13—H3C13	−118.4
C5—C4—C10—H1C10	177.7		

### Hydrogen-bond geometry (Å, °)

**Table:**

*D*—H···*A*	*D*—H	H···*A*	*D*···*A*	*D*—H···*A*
N2—HN2···OW^i^	1.00	1.83	2.763 (3)	154
N4—HN4···O2^ii^	1.00	2.00	2.858 (2)	143
OW—H1OW···O1	1.00	1.85	2.844 (2)	169
OW—H2OW···O1^iii^	1.00	1.81	2.796 (2)	169

Symmetry codes: (i) −*x*+1, −*y*+2, −*z*+1; (ii) −*x*+1, −*y*, −*z*; (iii) −*x*, −*y*+2, −*z*+1.
